# Development of bullous pemphigoid following radiation therapy combined with nivolumab for renal cell carcinoma: A case report of abscopal toxicities

**DOI:** 10.1097/MD.0000000000028199

**Published:** 2021-12-10

**Authors:** Linda My Huynh, Benjamin T. Bonebrake, Dominick J. DiMaio, Michael J. Baine, Benjamin A. Teply

**Affiliations:** a University of Nebraska Medical Center College of Medicine, Omaha, NE; b Department of Pathology and Microbiology, University of Nebraska Medical Center, Omaha, NE; c Department of Radiation Oncology, University of Nebraska Medical Center, Omaha, NE; d Department of Internal Medicine, Division of Hematology/Oncology, University of Nebraska Medical Center, Omaha, NE.

**Keywords:** abscopal effect, bullous pemphigoid, immunotherapy, radiation therapy, renal cell carcinoma

## Abstract

**Rationale:**

Concern for immune-related adverse events from immunotherapy and radiation therapy are well-documented; however, side effects are mostly mild to moderate. However, high-grade, potentially life-threatening adverse events are increasing. While case reports regarding immunotherapy-related bullous pemphigoid (BP) have been rising, only 1 has described BP following concomitant use of both nivolumab and radiation therapy (RT). For that patient, nivolumab was used for 10 weeks prior to RT and development of PB followed 7 weeks later. This case presents a patient who tolerated nivolumab well for 38 months prior to developing BP less than 2 weeks after completing RT.

**Patient concerns:**

We present the case of DH, a 67-year-old gentleman on nivolumab for metastatic renal cell carcinoma to the lung since May of 2017. Following progressing lung nodules, the patient had his nivolumab paused and completed a course of short-beam radiation therapy. After restarting nivolumab post-radiation, the patient presented with itchy rash and blisters on his arm, legs, and trunk.

**Diagnosis:**

DH consulted dermatology following development of rash and was diagnosed with bullous dermatosis, likely bullous pemphigoid. Bullous pemphigoid following concomitant nivolumab (OPDIVO), despite prior tolerance and no history of autoimmune disease, was confirmed by biopsy a month later.

**Interventions:**

Initial treatment was betamethasone 0.05% cream mixed 1:1 with powder to form paste applied twice daily. Given progressive symptoms and confirmatory biopsy of BP, nivolumab was held and 100 mg doxycycline and 80 mg prednisone daily was prescribed for a week, reduced to 60 mg during the second week.

**Outcomes:**

A week following discontinuation of nivolumab and beginning of doxycycline and prednisone, the blistering and rash was almost entirely resolved. Four months later, nivolumab was restarted and the patient continued low-dose tapering of prednisone until December. Since completing prednisone, the patient has shown no recurrence of bullous pemphigoid and has not developed any other immune-related adverse events to nivolumab upon rechallenge. Follow-up through October 2021 demonstrates the patient's sites of disease, both in- and out-field, have remained responsive to treatment.

**Lessons:**

Treating physicians should be aware of off-target effects of radiotherapy for oligoprogressive disease, which may include abscopal toxicities and the development of new immune-related adverse effects.

## 1 Introduction

Immune checkpoint inhibitors such as anti-programmed death-1/programmed death ligand-1 have emerged as an effective therapy for a variety of advanced malignancies. However, immune-related adverse events (irAE) are commonly reported and highly dependent on agent, dose, and exposure time.^[1]^

Adding another dimension to these irAEs is the concomitant use of radiation therapy (RT). While RT has been traditionally viewed to be immunosuppressive, radiation-induced activation of the immune system has been increasingly recognized.^[2]^ In this regard, these abscopal effects have been leveraged as a strategy to amplify the host's anti-tumor immune response during treatment. While the exact underlying mechanism of this abscopal effect remains unclear, it is speculated that it is the enhanced tumor antigen presentation and associated improved anti-tumor immune education afforded by RT that is responsible for the abscopal effect.

Despite these positive abscopal effects in reducing cancer burden, the concern for additional irAEs remains. Cutaneous side effects have been noted following RT, but are often limited to radiation dermatitis with associated erythema, pruritis, and occasionally, focal desquamation.^[2]^ Similarly, the use of immunotherapies have also been correlated with dermatologic irAEs. While most of these side effects are mild to moderate in severity, some manifestations can progress to high-grade and potentially life-threatening situations - such as bullous pemphigoid.^[3,4]^ Since 2015, more than 40 cases of immunotherapy-related BP have been reported, involving either the skin or mucous membrane. Of these case reports, only 1 described the development of BP following concomitant use of both nivolumab and RT.^[5]^

The increasing prevalence of these conditions not only raise concern for more serious toxicities, but also suggests the need for further investigation to elucidate possible mechanisms, risk factors, and management strategies for patients undergoing multi-modal treatment. In this report, we explore the case of a 67-year-old gentleman who developed BP shortly following RT to a lung metastasis, despite reporting no irAEs on nivolumab for 38 months of prior treatment.

## 2 Case presentation

We present the case of a 67-year-old gentleman with no history of autoimmune disease, was diagnosed with clear cell renal cell carcinoma (RCC) stage T3b, Fuhrman grade 4/4, and underwent a right radical nephrectomy, right adrenalectomy, and vena cava tumor thrombectomy in November 2016. After a follow-up computed tomography in March 2017 demonstrated multiple lung nodules, the patient underwent a left upper lobe wedge resection in April 2017 which confirmed metastatic renal cell carcinoma to lung. Nivolumab 240 mg in sodium chloride 0.9% 100 mL infusion every 2 weeks was started in May of 2017 progressing to 480 mg nivolumab every 4 weeks on May 1, 2018. The patient reported no irAEs with nivolumab for the following 38 months.

In April 2020, computed tomography demonstrated oligoprogressive disease with increase in size of 2 lung nodules, but otherwise stable disease. The patient was referred to radiation oncology and was treated with stereotactic body radiation therapy 5000 cGy in 5 fractions to both progressive lesions. Maximum dose to skin was 1481 cGy with less than 5 cc of skin receiving 1000 cGy or more (200 cGy per fraction). The associated dose to skin was minimal with only 5 cc of skin receiving 1000 cGy or more. Radiation was well-tolerated and completed on May 22, 2020.

After completion of short-beam radiation therapy, nivolumab 480 mg in sodium chloride 0.9% 100 mL infusion was continued on schedule with the next infusion provided on May 26, 2020. Following this, the patient developed diffuse, mildly pruritic skin lesions with blisters and presented to dermatology 1 week later with multiple erythematous bullae on the trunk, as well as the upper and lower extremities. Some bullae coalesced into crusted plaques and others were hyperpigmented patches. At this presentation, less than 10% of the patient's body showed involvement characteristic of bullous dermatosis and he was appropriately characterized to have mild to moderate cutaneous toxicity. A topic steroid (betamethasone 0.05% cream) was prescribed to manage the rash and nivolumab was continued accordingly.

Following the next 480 mg nivolumab in sodium chloride 0.9% 100 mL infusion 1 month later, the patient's bullous dermatosis worsened, with progressive involvement of the upper and lower extremities. The patient presented with progressive foot involvement and linear erosions of the face. Additionally, the patient had developed edema of the lower extremities resulting in a follow-up with dermatology on July 6 and punch biopsy on July 14.

**Figure 1:**
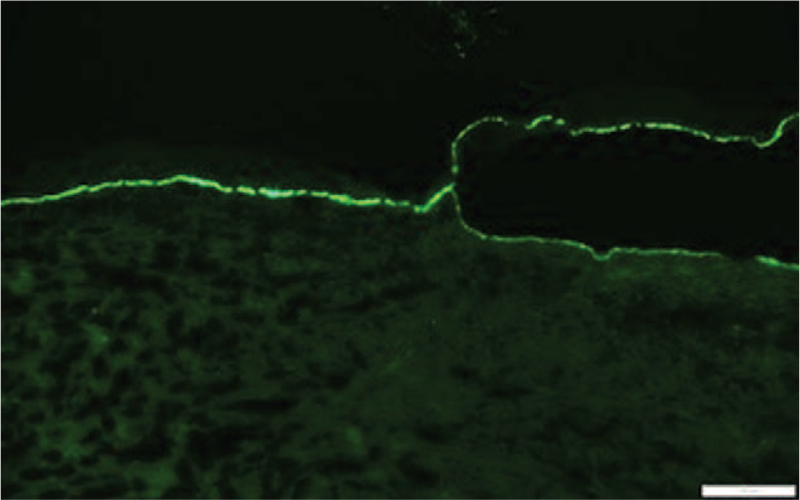
Subepidermal detachment of epidermis with band like inflammation within the papillary dermis (H&E, 40×).

**Figure 2:**
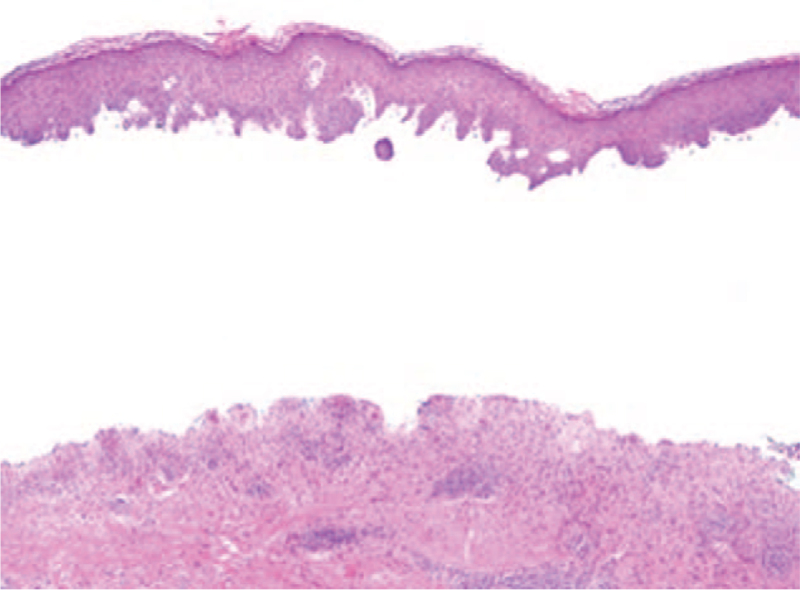
Inflammation within the papillary dermis consists of lymphocytes, large numbers of eosinophils and rare neutrophils (H&E, 200×).

**Figure 3:**
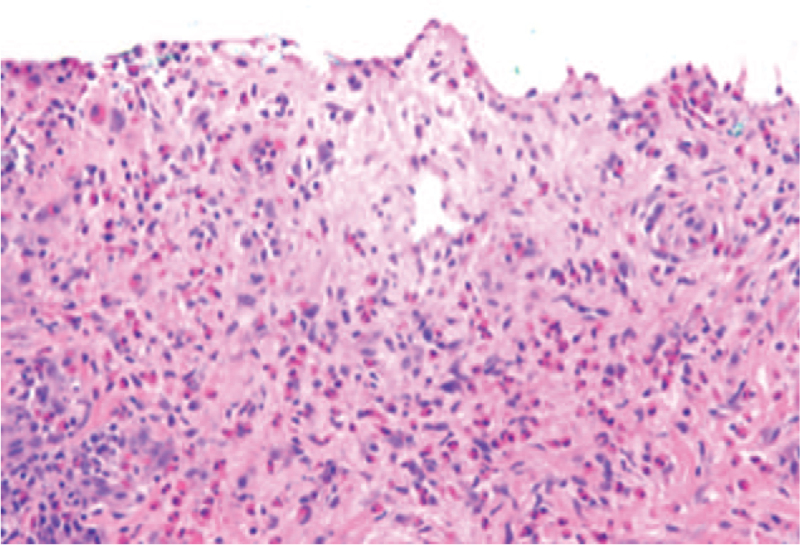
Linear staining for C3 along the dermal-epidermal junction. At the area of blister formation there is linear staining along both the roof and floor of the blister cavity. Similar staining was seen with IgG (direct immunofluorescence, 200×). IgG = immunoglobulin-G.

Sections of the biopsy demonstrated complete subepidermal detachment of the epidermis from the underlying dermis (Fig. 1). Within the papillary dermis there was a moderate band like inflammatory infiltrate composed of lymphocytes, a prominent number of eosinophils and rare neutrophils (Fig. 2). Direct immunofluorescence staining was performed on a separate skin biopsy and demonstrated linear staining for C3 and immunoglobulin-G along the basement membrane with no intercellular staining of the keratinocytes (Fig. 3). The histologic features in conjunction with the immunofluorescence staining supported a diagnosis of bullous pemphigoid.

Given the progressive symptoms despite topical corticosteroids and the biopsy proven development of bullous pemphigoid (BP), Nivolumab was held and the patient began treatment with 100 mg doxycycline and 80 mg prednisone daily for a week, reduced to 60 mg during the second week. On follow-up, the patient showed significant improvement and over the next 10 weeks was tapered down to 20 mg of prednisone. Four months following the initiation of treatment of BP, 480 mg nivolumab in sodium chloride 0.9% 146 mL infusion was restarted and the patient continued low-dose tapering of prednisone until December. Since completing the prednisone course, the patient has shown no recurrence of bullous pemphigoid and has not developed any other irAEs to nivolumab upon rechallenge. Follow-up through October of 2021 demonstrates the patient's sites of disease, both in- and out-field, have remained responsive to treatment.

## 3 Discussion

While cutaneous side effects are common and well-documented within the context of immunotherapy^[6,7]^ and radiation therapy alone,^[8]^ they are generally mild, localized, and do not result in life-threatening conditions (only 0.3%-1.3%).^[1]^ Nguyen et al^[8]^ published 29 cases of BP following RT monotherapy. Of the 29 patients included, 84% had received RT for breast cancer and BP was localized to irradiated sites in 25 patients.^[7]^ This is in stark contrast to the present case, wherein our patient developed a systemic BP less than 2 weeks following the addition of stereotactic radiation therapy to his previously well-tolerated regimen of nivolumab.

Tanita et al^[5]^ published a similar case on a patient with acral lentiginous melanoma who was treated with nivolumab and then intensity-modulated RT 10 weeks following nivolumab that also developed bullous pemphigoid. However, this case differs in several notable ways. First, their patient was treated with dacarbazine with interferon-B for 6 months prior to starting nivolumab. Second, the patient only demonstrated toleration of nivolumab treatment for 17 weeks compared to our patient's 38 months. Finally, the timeline differed as the development of bullous pemphigoid occurred 7 weeks after intensity-modulated RT irradiation compared to less than 2 weeks after short-beam radiation therapy irradiation in this present case.^[5]^ In our presented case, the temporal relationship between nivolumab dosing, radiotherapy, and development of BP is most consistent with a possible abscopal toxicity from radiotherapy.

While the mechanism by which immunotherapy and/or RT induces BP is currently unclear, it is reminiscent of the abscopal effect in RT.^[2]^ RT is traditionally considered immunosuppressive as it has direct and indirect cytotoxic effects via deoxyribonucleic acid damage and the generation of free radicals respectively, on irradiated immune cells. However, the abscopal effect suggests that RT may have immunostimulating properties as well. In this theory, RT is thought to create an antitumor immune response in the tumor microenvironment by inducing a release of anti-tumor cytokines and chemokines. These changes in the tumor microenvironment leads to chemoattraction of dendritic cells and cytotoxic T lymphocytes. This upregulation of the immune system response, along with radiation-induced susceptibility of tumor cells, thus results in shrinkage of tumors distant to the site of initial radiation. When combined with new immunotherapies and biologics, this synergistic effect has improved survival outcomes for various types of advanced carcinomas.^[2]^

Pouget et al^[9]^ suggests that radiation exposure results in systemic inflammation and an overactive immune response as the result of a chronic stress state. This is characterized by the production of damage-associated molecular patterns, which contribute to the upregulation of proinflammatory cytokines and an overactive systemic response. In this regard, it is ostensible that the RT-induced anti-tumor inflammatory state can also have negative consequences, as the balance between a controlled vs a pathologic immunogenic response is altered. This cascade has been correlated with several immune-related off-target effects such as radiation-related thyroid autoimmunity, diabetes mellitus, gastritis,^[10]^ and in this case, bullous pemphigoid. Further compounding this issue was the use of nivolumab in our patient. As nivolumab blocks programmed death-1/programmed death ligand-1, the addition of RT may have contributed to an overactive systemic response, leading to the development of off-target effects.^[8]^

Understanding immune-mediated adverse events is vital for providing adequate care to patients receiving concomitant RT and immunotherapies. While radiotherapy to oligoprogressive disease for patients on immunotherapy remains an attractive approach, treating physicians should be aware of the off-target effects, which may include the development of new immune-related adverse effects. Therefore, further studies are required to elucidate the mechanism by which RT and immunotherapy can induce BP and other negative abscopal effects.

## Author contributions

All authors participated in final approval of the version to be published and agree to be accountable for all aspects of the work in ensuring that questions related to the accuracy or integrity of any part of the work are appropriately investigated and resolved.

LMH: drafting the work; data acquisition and analysis.

BTB: drafting the work; data acquisition and analysis.

DJD: interpretation of data; revising work for intellectual content.

MJB: conception and design of work; interpretation of data; revising work for intellectual content.

BAT: conception and design of work; interpretation of data; revising work for intellectual content.

**Conceptualization:** Michael J. Baine, Benjamin A. Teply.

**Investigation:** Linda My Huynh, Benjamin T. Bonebrake, Michael J. Baine, Benjamin A. Teply.

**Methodology:** Dominick J. DiMaio, Michael J. Baine, Benjamin A. Teply.

**Project administration:** Michael J. Baine, Benjamin A. Teply.

**Resources:** Dominick J. DiMaio, Michael J. Baine, Benjamin A. Teply.

**Supervision:** Michael J. Baine, Benjamin A. Teply.

**Writing – original draft:** Linda My Huynh, Benjamin T. Bonebrake, Dominick J. DiMaio, Michael J. Baine, Benjamin A. Teply.

**Writing – review & editing:** Linda My Huynh, Benjamin T. Bonebrake, Dominick J. DiMaio, Michael J. Baine, Benjamin A. Teply.
